# Exhaled Metabolite Patterns to Identify Recent Asthma Exacerbations

**DOI:** 10.3390/metabo11120872

**Published:** 2021-12-15

**Authors:** Job J. M. H. van Bragt, Stefania Principe, Simone Hashimoto, D. Naomi Versteeg, Paul Brinkman, Susanne J. H. Vijverberg, Els J. M. Weersink, Nicola Scichilone, Anke H. Maitland-van der Zee

**Affiliations:** 1 Department of Respiratory Medicine, Amsterdam UMC, University of Amsterdam, 1105 AZ Amsterdam, The Netherlands; j.j.vanbragt@amsterdamumc.nl (J.J.M.H.v.B.); stefaniaprincipe90@gmail.com (S.P.); s.hashimoto@amsterdamumc.nl (S.H.); naomiversteeg@live.nl (D.N.V.); p.brinkman@amsterdamumc.nl (P.B.); s.j.vijverberg@amsterdamumc.nl (S.J.H.V.); e.j.weersink@amsterdamumc.nl (E.J.M.W.); 2 Dipartimento Universitario di Promozione della Salute, Materno Infantile, Medicina Interna e Specialistica di Eccellenza “G. D’Alessandro”(PROMISE) c/o Pneumologia, AOUP “Policlinico Paolo Giaccone”, University of Palermo, 90127 Palermo, Italy; nicola.scichilone@unipa.it

**Keywords:** exhaled breath, eNose, asthma, exacerbation

## Abstract

Asthma is a chronic respiratory disease that can lead to exacerbations, defined as acute episodes of worsening respiratory symptoms and lung function. Predicting the occurrence of these exacerbations is an important goal in asthma management. The measurement of exhaled breath by electronic nose (eNose) may allow for the monitoring of clinically unstable asthma and exacerbations. However, data on its ability to perform this is lacking. We aimed to evaluate whether eNose could identify patients that recently had asthma exacerbations. We performed a cross-sectional study, measuring exhaled breath using the SpiroNose in adults with a physician-reported diagnosis of asthma. Patients were randomly divided into a training (*n* = 252) and validation (*n* = 109) set. For the analysis of eNose signals, principal component (PC) and linear discriminant analysis (LDA) were performed. LDA, based on PC1-4, reliably discriminated between patients who had a recent exacerbation from those who had not (training receiver operating characteristic (ROC)–area under the curve (AUC) = 0.76,95% CI 0.69–0.82), (validation AUC = 0.76, 95% CI 0.64–0.87). Our study showed that, exhaled breath analysis using eNose could accurately identify asthma patients who recently had an exacerbation, and could indicate that asthma exacerbations have a specific exhaled breath pattern detectable by eNose.

## 1. Introduction

Asthma is a common chronic disease that affects approximately 5% to 10% of the global population with an increasing prevalence [1,2]. It is characterized by recurring respiratory symptoms (e.g., wheeze, shortness of breath, chest tightness and cough) associated with a variable and reversible airway inflammation [3]. Exacerbations, typically triggered by external agents (e.g., viral airway infections, exposure to allergens, smoke, exercise or stress) or poor adherence to medication [4,5], are acute episodes of worsening symptoms and lung function. Depending on the severity, the management of asthma exacerbations requires an increase of inhaler medication or a short-term course of additional oral corticosteroids [3,5]. They remain a key contributing factor to disease morbidity and increased health care utilization, leading to significantly higher health care costs, decreased quality of life and in severe cases, death [6,7,8]. The primary prevention of asthma exacerbations is therefore an important treatment goal.

Considered now to be key in defining the severity of the disease, exacerbations and their prevention is an important metric to measure the success of asthma treatments. Currently, biomarkers such as sputum eosinophils have shown some utility in monitoring and assessing disease severity, but are not predictive, and are often complex and invasive [9]. Instead, past exacerbations remain the most important risk factor for future exacerbations but can only indicate clinical instability [10]. Therefore, there remains an urgent need for predictive biomarkers.

The analysis of metabolites in exhaled breath using an electronic nose (eNose) is a non-invasive technique that shows promise. It detects patterns of volatile organic compounds (VOCs) that may reflect (patho)physiological changes related to chronic airway inflammation [11]. Previous studies have shown its ability to discriminate between clinically stable and unstable episodes of asthma, [12] and predict steroid responsiveness in patients [13]. Furthermore, eNose measurements were able to identify different phenotypes in patients with severe asthma in a combined cohort of asthma and COPD patients [14,15], where they also demonstrated an ability to detect recent exacerbations in COPD patients [14,15].

The analysis of breath profiles using a quick and non-invasive technique such as eNose, may allow for the identification of asthma instability. However, to date, the supporting data is limited. Consequently, assessing whether it is possible to detect a recent asthma exacerbation using eNose would be an important first step in exploring its potential. We hypothesized that asthma patients with a recent exacerbation display a different exhaled breath profile compared to those without a recent exacerbation.

## 2. Results

### 2.1. Baseline Characteristics

In total 361 asthma patients were included in the analysis; 252 were randomly allocated to the training dataset and 109 to the validation dataset. The number of patients with exacerbations was 41 in the training set (16.3%) and 11 (10.1%) in the validation set. Baseline patient characteristics of both the training and the validation dataset are shown in Table 1 and Table 2. Compared to patients without exacerbations, patients who had exacerbations, reported an increased use of antibiotics for the worsening of respiratory symptoms within the three months prior to eNose measurement (41.5% vs. 2.8%). In addition, maintenance use of inhaled corticosteroids (ICS) was also higher in this group (90.2% vs. 70.1%). Lung function values were similar across both groups, as was the percentage of patients with atopy (70.1% in exacerbation group vs. 71.6% without exacerbation group). Other clinical factors, such as reported asthma symptoms (based on the asthma control questionnaire (ACQ) score), age and BMI were also comparable. In the exacerbation group there were no current smokers compared to 6.2% in the no exacerbation cohort, however the percentage of never-smokers was similar (63.4% and 69.7%), as was the number of pack-years (4.8, IQR; 2.0–8.8 and 5.0, IQR; 2.0–8.0). 

### 2.2. Discriminant Analysis

**Figure 1:**
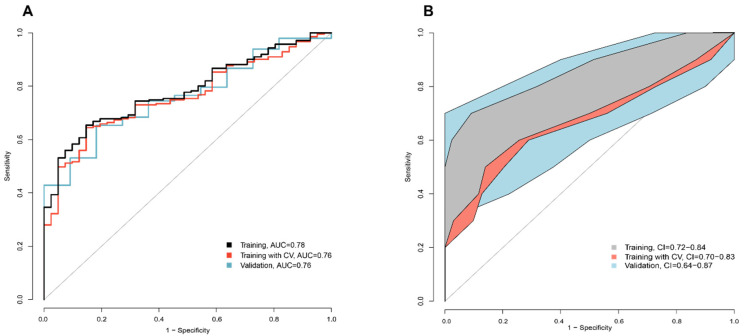
(**A**) ROC curves for the discrimination between asthma patients with recent exacerbations and without exacerbations; (**B**) 95%-confidence intervals of ROC curves.

Linear discriminant analysis (LDA) based on these relevant principal components (PC) showed an ability for eNose sensor signals to discriminate between patients who had had an exacerbation from those who had not (ROC-AUC = 0.78 (95%-CI: 0.72–0.84)). After cross-validation, this resulted in a ROC-AUC = 0.76 (95%-CI: 0.69–0.82). A similar accuracy was observed in the validation dataset (ROC-AUC = 0.76 (95%-CI: 0.64–0.87)), See Figure 1. 

### 2.3. Sensitivity Analysis

The ROC analysis was repeated while omitting patients with: (1) a recent history of antibiotic use for acute worsening of respiratory symptoms; (2) current smokers at the time of measuring or (3) those who did not use inhaled corticosteroids as maintenance treatment. Similar results were seen for the discrimination in both the training and validation datasets (ROC-AUC = 0.81, when omitting current smokers training ROC-AUC = 0.77 and when only including patients on maintenance ICS training ROC-AUC = 0.83). All AUC results can be found in Table 3. 

## 3. Discussion

This study showed that the measurement of exhaled breath patterns by eNose could accurately discriminate between asthma patients with a recent exacerbation and those without. These results were validated and confirmed in a validation set. A sensitivity analysis showed that despite recent use of antibiotics, smoking history or ICS use, the discriminative accuracy was preserved. Our results suggest that there is a difference in exhaled metabolites between asthma patients who have had a recent exacerbation in the previous 3 months and those who have not. This might be of interest for the monitoring of disease stability. To our knowledge, this is the first study that proved the ability of exhaled breath patterns measured with eNose to detect recent asthma exacerbations in adult patients.

In several studies, the predictive properties of exhaled VOCs associated with asthma exacerbations have been explored in children by analyzing gas chromatography coupled with mass spectrometry (GC-MS) data [16,17,18]. A classification model consisting of seven VOCs (three aldehydes, one cyclic alkane, one ketone, one aromatic VOC and one unidentified VOC) was able to correctly predict up to 88% of asthma exacerbations, but the accuracy decreased when the sample was collected further from when the exacerbation first occurred [16]. In our study, exacerbations were defined based on the recent OCS use for acute worsening in respiratory symptoms within the three months prior to eNose measurement. However, it remains unclear what the correct timing on performing such measurements should be and is likely that that VOC patterns during asthma exacerbations vary at different time points. Moreover, our results are in line and strengthen a previous study, in which breath profiles measured by eNose correctly classified clinically stable and unstable episodes of asthma where unstable asthma was defined according to inhaled steroid withdrawal [19].

A recent study, looking into the differences in exhaled breath patterns after a viral challenge in asthma patients, found a detectable change in fluctuations in eNose signals after viral infection. These changes appeared to commence before the expression of viral symptoms [20]. Since viral infections can easily be one of the triggers for asthma exacerbations, this might suggest a potential role of exhaled breath analysis in prediction of exacerbations.

The, hereby, presented results are in line with a similar study conducted by our group in COPD patients. They demonstrated that eNose analysis was able to discriminate between COPD patients who had experienced an exacerbation and those who had not. However, the overall accuracy was better for COPD patients compared to asthma patients presented in the current study (ROC-AUC in validation dataset = 0.98 vs. 0.76, respectively) [19].

These differences maybe attributable to several reasons; firstly, VOCs and consequently exhaled breath patterns measured by eNose, could be different in asthma exacerbations compared to COPD exacerbations due different pathologic triggers. It has been demonstrated that allergen and viral exposure can trigger asthma exacerbations, while, bacterial infections play less of a role [21]. For instance, the use of adjunctive antimicrobial treatment for asthma exacerbations has been shown to lead to worse outcomes (longer hospitalization, increased hospital costs and risk of treatment failure) when compared to patients treated only with when OCS [22]. Consequently, only COPD guidelines mention bacterial causes and relate them to an increase in neutro- and eosinophilic inflammation [23,24]. Previous eNose-studies suggest that the differences detected using eNose may reflect specific inflammatory processes related to different phenotypes in chronic airways diseases [15]. In particular, exacerbations in asthma and COPD have been related to eosinophilia whereas neutrophilia is more often only associated with COPD [25]. Furthermore, a previous study found different eNose-driven clusters in asthma patients with differences according to neutrophilia, eosinophilia and OCS use [26].

Another reason for the differences in accuracy maybe be related to the influence of smoking exposure on exhaled breath patterns. Oxidative stress induced by exposure to smoking is an important risk-factor for disease pathogenesis in COPD patients [27]. Asthma patients with a smoking history of ≥10 pack-years were excluded from our analysis and the proportion of non-smokers in our study is much higher than that of current smokers, thus, in our asthma population the influence of oxidative stress is expected to be much smaller. However, the sensitivity analyses in which current smokers were excluded did not show large differences in accuracy. This supports previous results where smoking was found not to be a confounding factor that affects eNose results [28].

In our study we found a differences in recent exposure to antibiotics for the treatment of acute respiratory symptoms between the exacerbation and no-exacerbation groups. Respiratory infections impact metabolism and exhaled breath VOCs that can result from systemic effects [13], thus residual effects might be reflected in the eNose signals. VOCs could also be a result of drug metabolism and reflect antibiotic drugs or OCS use, although a clear distinction between exacerbations with or without respiratory infections is difficult to quantify. Additionally, it has been suggested that eNose analysis could predict systemic steroid responsiveness [29], therefore steroid use is expected to influence eNose signals. Drug monitoring via exhaled breath is an emerging field [30], future research should focus on disentangling the exacerbation itself from a possible residual medication effect.

The strengths of the current study are the relatively large sample size and the heterogeneous population of the asthma cohort, which has been recruited from centers of primary, secondary and tertiary care, reflecting a real-life asthma population. The use of (internal-) cross-validation and splitting our dataset to allow validation of the training discriminant model in a separate dataset, support the validity of the obtained results. Furthermore, current results are in line with our previous similar study in a COPD population, however with lower accuracy.

This study has several limitations; defining exacerbations is challenging. It is possible that the definition used for exacerbations in our study (current or previous treatment with OCS for acute worsening of respiratory symptoms) is not really representative of all patients with exacerbations even though, in most clinical trials, exacerbations are defined as a significant deterioration of asthma signaled by the need for a systemic corticosteroid course (≥3 days) and/or hospitalization for asthma and/or emergency room attendance for asthma [31,32,33]. In our data, we do not have information regarding the use of short acting bronchodilators as rescue medication and/or the necessity of hospital admissions, as stated in international guidelines [4], therefore we used a similar approach in defining asthma exacerbations as has been used in several previous studies [34]. Misclassification may have occurred, most probably leading to underestimation of the outcome. In addition, we were not able to exclude whether some of the patients received OCS for reasons other than an asthma exacerbation. However, Pont et al. [35] determined the reliability of identifying asthma exacerbation episodes based on asthma medications in the general practice, in particular, one of the definitions of asthma exacerbation was related to the assumption of OCS use for a short-time period. 

Another limitation of our study is related to the lack of information regarding the timing of the exacerbations. In the current study patients only reported whether they used OCS for worsening of respiratory symptoms within 3 months prior measurement (yes/no) and information on specific timing was not available. We know from previous studies [36], that past exacerbations, in particular, those that require OCS, are predictors for future exacerbations. Therefore, exhaled VOCs in regard to the time of the exacerbation should be topic of future (longitudinal) studies. Additionally, our study information about the occurrence of exacerbations was patient-reported. Therefore, it could have been subjected to recall bias. However, considering that our definition of exacerbation was within a short time frame (< 3 months) and the potential severity of symptoms often associated with an exacerbation, it is likely that recall bias was limited. Furthermore, previous studies used a similar, self-reported definition of exacerbation [36,37].

A further limitation is that eNose identifies breath patterns, therefore a mixture of VOCs, rather than specific volatile compounds. Nonetheless, this makes eNose breath profiles identifiable as a pattern of biomarkers mostly focused on giving a particular probability of the presence/absence of a clinical condition [38]. Specific VOCs can be analyzed with methods such as gas chromatography mass spectrometry (GC-MS), which can be useful for identifying specific target VOCs and linking them to possible pathophysiological pathways [39]. However, this technique is significantly more labor-intensive and requires highly trained personnel. 

Our findings support future studies with more emphasis on the timing of the exacerbations and the relation of fluctuations of exhaled VOCs to (in)stability of asthma patients. Differences in, for example, diet and comorbidities could influence exhaled breath composition, and due to these (probably still unknown) confounding signals it will be difficult to achieve a high enough accuracy in diagnosing. Patterns that diverge from baseline measurements could indicate instability and with this study we show that eNose measurements are indeed able to detect possible changes related to exacerbations. eNose is a non-invasive and easy to use technique, that could be beneficial for future patient monitoring. It could be used for assessing treatment efficacy or as an early-warning for the onset of exacerbations, enabling timely treatment. Furthermore, as past exacerbations are a risk factor for future exacerbations in patients with asthma, our results suggest a role in identifying patients at risk of future exacerbations and optimizing treatment. 

## 4. Materials and Methods

### 4.1. Population

This was an exploratory cross-sectional study and data was retrieved between December 2015 and January 2017 from the multicenter BreathCloud database, which contains exhaled breath and clinical data of patients with respiratory diseases (asthma, COPD, lung cancer) and healthy controls [15,19,28,40,41]. BreathCloud data was collected during routine outpatient visits in primary, secondary and tertiary care centers. The medical ethical review board of Amsterdam UMC, location AMC provided a waiver for ethical approval due to the non-invasive nature of the measurement, nonetheless written informed consent was obtained from each participant before enrolment. In this study we included adult (>18 years) participants with a physician diagnosis of asthma. Participants with a diagnosis of COPD and/or lung cancer, smokers or ex-smokers with ≥10 packyears and participants who were currently using antibiotics for acute respiratory symptoms were excluded before analysis.

### 4.2. Outcomes

Recent exacerbations were defined as an acute worsening of respiratory symptoms, which required the use of oral corticosteroids anytime during 3 months prior to or during the eNose measurement [34].

### 4.3. Exhaled Breath Analysis

Volatile organic compounds in exhaled breath were measured with the SpiroNose (Amsterdam UMC, Amsterdam, the Netherlands). This eNose uses different cross-reactive metal oxide semiconducting sensors (MOS), grouped in eight arrays of three or four sensors; four arrays are used for measuring exhaled breath and four arrays are used to measure ambient air VOCs. The sensor material, mostly composed of Tin dioxide (SnO_2_), is printed on electrodes using an alumina substrate and those sensors (Figaro Engineering Inc., Osaka, Japan) have been selected for their good stability and performance [42]. In total seven different sensors are used, and they have (cross-reactive) high sensitivity to VOCs, ammonia, H_2_S, butane, propane, methane, hydrogen, ethanol, trimethylamine, methylmercaptan and solvent vapors in the 1–10.000 ppm range. The sensors are heated and acquire semiconducting characteristics as a result. Signals are derived from changes in resistance (and therefore conductance) in the sensors due to interactions with oxidizing/reducing gases.

**Figure 2:**
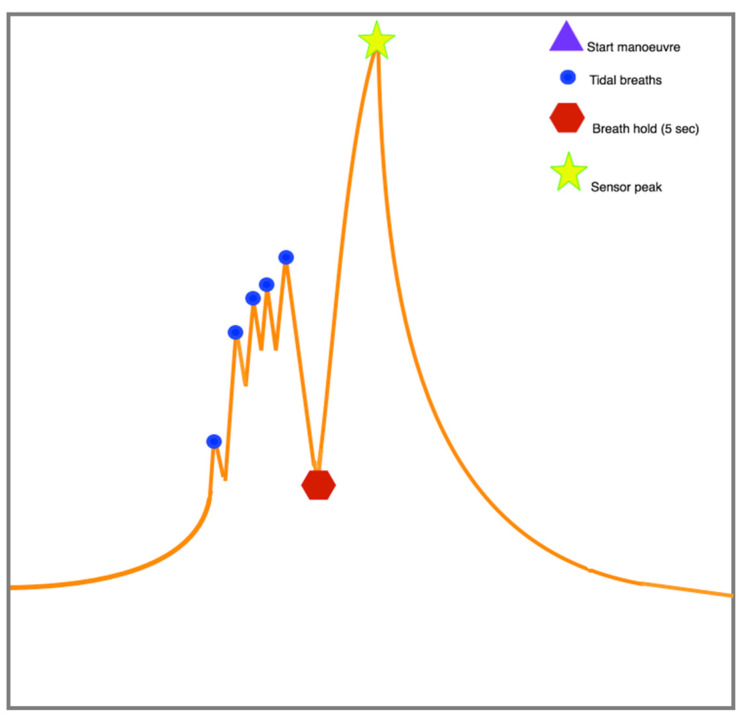
eNose maneuver. Adapted from Mahmoud I. Abdel-Aziz et al. [40].

The study (exhalation) maneuver existed of five tidal breaths followed by a single full inspiration, a 5 s breath hold and a slow maximal expiration to residual volume. Sensor data was captured in real-time and directly stored on the BreathCloud server (Figure 2). Pre-processing of sensor signal data has been described previously [14] and consisted of advanced signal processing where the Eigen frequencies were removed, the signals were passed through a Butterworth filter and linear trends were removed. Ambient air correction was based on cross correlation and alveolar gradient calculation and quality control of sensor stability was verified monthly by using a test gas (Lindegas). Sensor values were normalized to the most stable sensor (sensor 2) and the subsequent sensor peak value and the ratio between sensor peak and breath-hold trough (13 variables in total) were used for further (statistical) analysis.

### 4.4. Statistical Analysis

Statistical analyses was performed in R studio (Rstudio Inc., Boston, MA, USA) using R version 4.0.5 (The R Foundation for Statistical Computing, Vienna, Austria, with packages: tidyverse, caret, pROC and MASS). Between groups comparison used independent t-tests, Wilcoxon rank-sum tests, and Pearson’s chi-squared tests as appropriate with a significance level *p* < 0.05. Multiple group comparisons (>2 groups) used one-way ANOVA, Kruskall–Wallis or Chi-squared tests as appropriate. 

The included asthma patients were divided into a training (70%) and validation (30%) dataset, similar to a previous similar study [19]. To cope with cross-reactivity of the sensors, and thus multicollinearity of the sensor signals, the sensor signals in the training dataset were reconstructed into their principal components (PC). These PCs, if their Eigen value was ≥1.0 [43] were then used in a linear discriminant analysis (LDA). The resulting discriminant scores were used to create receiver-operating-characteristic (ROC) curves and calculate the area under the curve (AUC) to assess the ability to discriminate between patients who exacerbated and those who did not. Leave-one-out cross-validation verified internal validity and AUC confidence intervals were calculated with 10,000 bootstrap iterations. The external validity was assessed with the validation dataset and used the rotation matrix standardization parameters of the training PCs to calculate validation sensor signal principal components. The training LDA model was then used to calculate discriminant scores based on validation PCs and these scores were subsequently used to create ROC curves and calculate AUCs.

### 4.5. Sensitivity Analysis

Variables that differ between the exacerbation and no exacerbation groups and are expected to have a potential influence on eNose sensor signals were used in extra sensitivity analysis. To assess the effect of these variables on the outcome, the ROC analyses were repeated while omitting study participants with these characteristics from the analysis.

## 5. Conclusions

Exhaled breath analysis by eNose can accurately discriminate between asthma patients who have experienced an exacerbation and were treated with OCS during the previous 3 months and those who did not. The current study suggests that asthma patients during or after a recent exacerbation have a specific exhaled molecular ‘breathprint’ that can be detected, thus suggesting a role in monitoring disease control and (in)stability for these patients.

## Figures and Tables

**Table 1:** Baseline characteristics of the training dataset.

	Total Training Dataset	Exacerbation	No Exacerbation
*n*	252	41	211
Age (years), mean (SD)	48.3 (18.1)	51.3 (16.1)	47.7 (18.5)
Gender, male (*n*, %),	89 (35.3)	9 (22.0)	80 (38.0)
BMI (kg/m^2^), mean (SD)	27.4 (6.5)	26.7 (5.0)	27.5 (6.8)
Smoking (never/ex/current), *n*	173/65/13	26/15/0	147/50/13
Pack-years, median (IQR)	5.0 (2.0–8.0)	4.8 (2.0–8.8)	5.0 (2.0–8.0)
Exacerbations *, *n* (%)	41 (16.3)	41 (100)	0 (0)
Previous AB use ^#^, *n* (%)	23 (9.1)	17 (41.5)	6 (2.8)
FEV_1_ as % of predicted, mean (SD)	88.4 (22.0)	88.4 (25.0)	87.2 (20.9)
Post-bronchodilator FEV_1_ as % of predicted, mean (SD)	90.1 (20.0)	88.0 (19.7)	92.7 (20.0)
FEV_1_/FVC as % of predicted, mean (SD)	86.7 (15.4)	87.8 (14.1)	86.7 (15.6)
Post-bronchodilator FEV_1_/FVC as % of predicted, mean (SD)	89.1 (14.6)	86.9 (14.5)	90.9 (14.4)
Blood eosinophils (cells·µL^−1^), median (IQR)	0.21 (0.09–0.42)	0.24 (0.11–0.47)	0.21 (0.09–0.42)
Blood neutrophils (cells·µL^−1^), median (IQR)	4.8 (3.5–6.0)	5.5 (4.3–7.5)	4.5 (3.4–6.2)
FeNO (ppb), median (IQR)	25.5 (15.0–41.0)	28.0 (17.5–39.5)	22.0 (13.0–47.5)
ACQ score, mean (SD)	1.8 (1.4)	1.8 (1.4)	1.7 (1.4)
ACQ-score > 1.5 (*n*, %)	130 (51.6)	23 (56.1)	98 (46.4)
Allergy ^†^, yes, *n* (%)	183 (72.6)	29 (70.1)	151 (71.6)
Use of ICS, yes, *n* (%)	213 (84.5)	37 (90.2)	176 (70.1)

* Defined as OCS for acute worsening of respiratory symptoms < 3 months prior measurement. ^#^ Defined as antibiotics for acute worsening of respiratory symptoms < 3 months prior measurement. ^†^ Self-reported. FEV_1_: forced expiratory volume in 1 s, FVC: forced vital capacity, FeNO: fraction of exhaled nitric oxide, AB: antibiotics, ACQ: asthma control questionnaire and ICS: inhaled corticosteroids.

**Table 2:** Baseline characteristics of the validation database.

	Total Validation Dataset	Exacerbation	No Exacerbation
*n*	109	11	98
Age (years), mean (SD)	48.2 (16.2)	55.7 (17.1)	47.3 (15.9)
Gender, male (*n*, %),	40 (36.7)	5 (45.5)	35 (35.7)
BMI (kg/m^2^), mean (SD)	28.3 (6.0)	29.5 (7.1)	28.1 (5.9)
Smoking (never/ex/current), *n*	85/14/10	10/1/0	75/13/10
Pack-years, median (IQR)	4.4 (2.5–8.5)	5.0 (5.0–5.0)	4.3 (2.3–8.8)
Exacerbations *, *n* (%)	11 (10.1)	11 (100)	0 (0)
Previous AB use ^#^, *n* (%)	5 (4.6)	3 (27.3)	2 (2.0)
FEV_1_ as % of predicted, mean (SD)	86.4 (21.0)	78.4 (25.7)	87.4 (20.3)
Post-bronchodilator FEV_1_ as % of predicted, mean (SD)	89.8 (17.6)	81.4 (19.7)	91.2 (17.0)
FEV_1_/FVC as % of predicted, mean (SD)	85.1 (14.9)	74.5 (24.0)	86.5 (13.0)
Post-bronchodilator FEV_1_/FVC as % of predicted, mean (SD)	87.0 (14.5)	75.5 (20.2)	88.9 (12.6)
Blood eosinophils (cells·µL^−1^), median (IQR)	0.22 (0.10–0.40)	0.28 (0.12–0.47)	0.21 (0.10–0.40)
Blood neutrophils (cells·µL^−1^), median (IQR)	4.6 (3.6–6.1)	5.6 (4.5–7.4)	4.5 (3.6–5.8)
FeNO (ppb), median (IQR)	25.0 (15.3–39.0)	34.5 (24.0–39.0)	24.5 (14.8–39.0)
ACQ score, mean (SD)	1.8 (1.2)	2.3 (1.2)	1.8 (1.2)
ACQ-score > 1.5 (*n*, %)	65 (59.6)	9 (81.8)	56 (57.1)
Allergy ^†^, yes, *n* (%)	76 (69.7)	9 (81.8)	67 (68.4)
Use of ICS, yes, *n* (%)	95 (87.2)	10 (90.9)	85 (86.7)

* Defined as OCS for acute worsening of respiratory symptoms < 3 months prior measurement. ^#^ Defined as antibiotics for acute worsening of respiratory symptoms < 3 months prior measurement. ^†^ Self-reported. FEV_1_: forced expiratory volume in 1 s, FVC: forced vital capacity, FeNO: fraction of exhaled nitric oxide, AB: antibiotics, ACQ: asthma control questionnaire and ICS: inhaled corticosteroids.

**Table 3:** Sensitivity analysis of the ability to discriminate between recent exacerbation and no-exacerbation in different subpopulation.

	Training AUC	Cross-Validated AUC	Validation AUC
All asthma patients (*n =* 361)	0.78 (0.72–0.84)	0.76 (0.70–0.83)	0.76 (0.64–0.87)
Without antibiotics users (*n =* 333) *	0.81 (0.75–0.87)	0.79 (0.72–0.85)	0.81 (0.70–0.92)
Without current smokers (*n =* 338)	0.77 (0.71–0.84)	0.75 (0.68–0.82)	0.74 (0.60–0.87)
Only with patients who use maintenance ICS (*n =* 308)	0.83 (0.78–0.89)	0.81 (0.76–0.87)	0.84 (0.75–0.93)

* Defined as use of antibiotics for acute worsening of respiratory symptoms < 3 months prior measurement. AUC: area under the curve and ICS: inhaled corticosteroids. Data is shown as AUC with 95% confidence interval.

## Data Availability

The data presented in this study are available on request from the corresponding author. The data are no publicly available due to privacy.
